# What is a “Good Life”: Protocol for a qualitative study to explore the viewpoint of older persons

**DOI:** 10.1371/journal.pone.0261741

**Published:** 2021-12-23

**Authors:** Hervé Michel, Hélène Prévôt-Huille, Raphaël Koster, Fiona Ecarnot, Zoé Grange, Stéphane Sanchez

**Affiliations:** 1 MADoPA, Troyes, France; 2 Department of Cardiology, University Hospital Besançon, Besançon, France; 3 EA3920, University of Burgundy Franche-Comté, Besancon, France; 4 Public Health, Department on Performance and Public Health, Champagne Sud Hospital, Troyes, France; 5 EA3797-Viellissement Fragilité, URCA, Reims, France; Illawarra Shoalhaven Local Health District, AUSTRALIA

## Abstract

**Introduction:**

Over the last fifteen years, Living Labs have been on the rise in Europe to bridge the gap between service providers, and the needs of end-users, and to speed up innovation, particularly in the field of healthcare and ageing. Ageing tends to be considered by institutions as a set of risks to be managed for older persons, illustrated in particular via the concepts of “ageing well” or “successful ageing”. In this context, this project aims to define the meaning and the conditions for a good life from the point of view of older persons themselves, thereby improving institutions’ recognition and support of older persons’ ways of living well, rather than imposing a general definition of “successful ageing” based on functional capacity.

**Methods and analysis:**

This qualitative study is designed as an action research underpinned by a Living-Lab approach to co-creation. The aims are to: define the conditions for a good life as accurately as possible with older persons (Step 1); share these findings with different healthcare and service providers to adjust existing services or create new ones (Step 2); and disseminate them more broadly within the regions under study and across the scientific community (Step 3). During Step 1, the features of a “good life” will be analysed in a socio-anthropological study based on semi-directed interviews and observations made in the homes of 70 elderly people living in a wide range of accommodation types and regions. In accordance with French legislation, and as confirmed by our formal Ethics Committee, this study does not require approval. The dissemination stage is integrated into the design of this action research, and notably will provide for the appropriation of research findings by the partners of this study, by setting up creativity sessions (Step 2) and by sharing the general findings through panel discussions bringing together regional and national stakeholders (Step 3).

## Introduction

By 2060, one in three people will be over 65 in Europe [1]. This simple demographic statistic raises questions surrounding the financial and organisational sustainability of European systems of social security, as well as care and support policies and programmes for older persons in hospitals, institutions and at home. This demographic transition also appears as an economic opportunity [2], while the “silver economy” or senior market (including consumption goods, prevention of autonomy loss, home care support) is regarded as a market with strong potential for growth and innovation, especially in view of its association with the development of new technologies (artificial intelligence (AI), big data, digitalisation) and customised medicine.

Many Living Labs and Living-Lab networks have emerged in Europe with the support of the European Union [3], EIT Health (https://eithealth.eu/) or at the initiative of national, local public and private actors. The term “Living lab” generally refers to the involvement of multiple stakeholders, including users, in the exploration, co-creation and evaluation of innovations within a realistic setting [4]. The aim is to adjust the offer of services to the end-users’ real needs and to support developments in innovation, involving older persons at the different stages of defining needs and developing solutions.

The current project was launched by a Living Lab in partnership with healthcare and service providers. It is based on a user-centred methodology characterised by 3 main approaches:

1. An innovation approach: not for users but with or by users (regarded as actors in their own right) [3].
2. A socio-anthropological approach to the analysis of needs in real-life [5], making it possible to observe and mobilise older persons in their living spaces, in relation to the people who support them in their daily life.
3. An approach to ageing as a social situation, as an evolution of ways of life structured by problems, concerns, as well as assets [6] and motivations [5], which need to be defined with older persons, rather than exclusively viewing ageing as an accumulation of risks that need to be managed.

### Scientific context

Political and health institutions have developed a wide range of concepts and tools to define the framework for ageing well, within a perspective of risk management (linked to older persons’ health and autonomy), and/or citizen engagement: active ageing [7, 8], healthy ageing [9], age-friendly cities [10], ageing in place [11–13], etc.

Compared to the institutional literature, this project does not aim to focus on the risks associated with ageing that form the basis for these institutions’ recommendations for ageing well. In this project, older persons are invited to share both their concerns and their motivations. More broadly, while the institutional literature focuses on ageing and the conditions for promoting successful ageing, the present project focuses on daily life and older persons’ conditions for living a good life, thus coinciding with the more recent developments in social science and humanities research.

In the field of gerontology, there is a vast amount of literature covering the multidimensional notion of ageing well and successful ageing [14]. First, following a prescriptive approach, defining the way in which individuals should age, and then progressively moving towards a more comprehensive approach, based on the inclusion of the perceptions older persons have of their own lives [15]. A literature review on successful ageing [16] highlights the various calls by researchers to “take into account the criteria for successful ageing as defined by older persons”, “what matters to older persons” and “integrating the perceptions of older persons about ageing in the definition of successful ageing”.

Literature on the multidimensional concept of quality of life has also shown the importance of adopting the point of view of older persons to define quality of life [17] and having specific tools to measure it [18], particularly in the context of “ageing in place” [19].

Work on subjective well-being applied to older persons has also gained ground in the field of psychology, making a distinction between “evaluative wellbeing (or life satisfaction), hedonic wellbeing (feelings of happiness, sadness, anger, stress and pain) and eudemonic wellbeing (sense of purpose and meaning in life)” [20].

Finally, a strand in sociology called micro-sociology [21] or socio-anthropology focuses on a comprehensive approach to the point of view of older persons. This type of approach has particularly gained ground in the sociology of ageing over the last twenty years, shedding new light on life trajectories and key moments in daily life [22], identity negotiation in old age [23, 24], “*culture du domicile*” [the being-at-home culture] [25], adaptation strategies to ageing by disengagement (concept of *déprise*) [23, 26] or selecting activities, objects, relationships and places that are meaningful to people [27], and on services offered to older persons [28–30]. In this perspective, the meaning of a good life for older persons and their quality of life both appear as a subjective appreciation in a social situation.

The present project, entitled “A Good Life: the point of view of older persons” is based on a socio-anthropological approach to the evolution of the older person’s ways of life. It aims to engage with a variety of institutional and scientific literature focused on ageing well, as well as on the wellbeing and quality of life of older adults.

## Methods

### Aims

Within a more global Living Lab approach [4, 5, 31–34], this project aims to create the conditions for healthcare and service providers to appropriate this knowledge in order to adjust their offer of services to the real needs of older persons.

The meaning of a good life will be defined with older persons living in a variety of accommodation types and regions, mainly through the following:

- The identification of existing activities and relationships that support and motivate older persons (their current meaningful activities and logics of action). These questions have previously been the topic of specific research at national level for the CNSA (French National Solidarity Fund for Autonomy), retirement funds, the Ministry of Economy and Finance and recently covered in a scientific publication [29].
- Their appreciation of conditions that are essential for quality of life and/or services, by targeting both unmet or unrecognised needs, as well as renunciations and opportunities related to the set-up of home care support plans or changes in location or accommodation type, and finally the ways in which people feel at home (in their accommodation and/or local environment).
- The comparison of findings between older persons living at home, in independent-living facilities, nursing homes and atypical accommodation types.

Therefore, this project aims to refine the offer of services as regards their ability to support and promote meaningful activities for older persons, while improving their quality of life in light of the main conditions and items defined by older persons themselves.

The objectives of this study are therefore to:

- Refine a typology of meaningful activities and logics of action in older persons.
- Define conditions and essential features for quality of life and/or services from the point of view of older persons.
- Create optimal conditions for healthcare and service provider partners in this study to appropriate findings by diversifying the channels for sharing and valuing these findings (operational reports, creativity sessions, panel discussions in regions, scientific publications).
- Contribute to the adjustment of the offer of services in nursing homes, independent-living facilities and for community dwellers living at home.

In accordance with French legislation, this study does not require approval from a formal Ethics Committee. The Ethics Committee CPP Est I (Dijon, France) confirmed that this protocol does not require formal approval.

### Project structure

This project is designed as an action research, committed to highlighting and creating supportive conditions that favour the expression of what matters to older persons.

More precisely, it is based on a Living Lab approach, which aims to: define the conditions for a good life with older persons as accurately as possible (Step 1); create optimal conditions for the appropriation of these findings by healthcare and service providers who are partners in this study (Step 2); and disseminate these findings in the regions under study and within the scientific community (Step 3).

#### Step 1: Field study

This socio-anthropological study will look at ageing as a social experience lived by the individual within a collective context.

The project “A Good Life: The point of view of older persons” will be implemented using interviews conducted with older persons, and as far as possible, with their carers and the professionals supporting them on a daily basis. These interviews will enable us to understand the point of view of older persons within the social and care networks that surround them, while also enabling us to observe their living environments, private and shared spaces within institutions and public spaces.

#### Step 1 interviews: Inclusion criteria

This step is based on 70 interviews and observations with older people, aged over 60 years, able to express consent, and living in a variety of accommodation types (nursing homes, independent-living facilities, at home, atypical forms of accommodation, such as intergenerational flat/house sharing, cohabitation with family members, cooperative housing, host family, medicalised or modular cottages, feeling at home elsewhere (nomadic retiree or traveller)) and areas (urban, semi-urban, rural).

Interviews are grouped according to these inclusion criteria as follows:

- 20 interviews with dependant elderly people in nursing homes
- 20 interviews with autonomous elderly people in independent-living facilities
- 20 interviews with autonomous elderly people living at home
- 10 interviews with dependant and autonomous elderly who chose to live in an atypical type of accommodation (complementary and exploratory panel)

Depending on the panels, older persons are recruited either directly by the partners of this study, or indirectly by intermediaries (associations, clubs, acquaintances, etc.) according to the specific sub-criteria of each panel, as defined by the research team, the aim being to reach as wide a range of profiles as possible.

#### Step 1: Study implementation

Volunteers will be interviewed by semi-structured interviews using a common interview grid. Interviewers (4) are part of the MADoPA Living Lab, doctors in political science, sociology or anthropology, and all have significant experience (between 7 and 20 years) with long interviews (1 to 2 hours). Each researcher is responsible for a specific panel and acts as an interviewer in other panels in order to gain a transversal understanding of the fieldwork.

The interviews will be recorded and filmed and will be thematically analysed after re-transcription. As far as possible, they will be preceded and/or followed by meetings and informal interviews with family (informal) carers or professional (formal) carers, neighbours, and other professionals with whom the older person has regular contact in their daily life (such as shopkeepers).

These interviews will be accompanied by natural observations made in the accommodation environment of older persons (private, shared and public spaces).

Thematic analysis of interviews will provide a summary based on five themes:

1. Daily activities and meaningful activities (those which motivate older persons because they are meaningful).
2. Social and care relationships, insofar as they support these central and meaningful activities (or not).
3. The significance given to “being at home”, paying particular attention to objects, living spaces and the importance of living in a given area (or not).
4. Adaptation to change (changes made to accommodation, changing accommodation, COVID-19, ageing), including the relationship to change (desired, consented, or imposed) and the assessment of the changes (constraints, renunciations, adaptation, new opportunities).
5. The uses and usefulness according to older persons of what the institutions call the “offer” of service, accommodation and technologies.

#### Step 1: Findings

Findings from Step 1 constitute the ‘matter’ to be appropriated by service and healthcare providers who are the partners of this study. By sharing these findings, we aim to illustrate the singular experiences of ageing (deliverables 1 and 2) and to develop a global approach to the conditions needed for a good life according to older persons (deliverables 3 and 4). Four main deliverables are expected. A summary of the 70 case studies (deliverable 1) with video supports from the filmed interviews (deliverable 2), a typology of logics of action among older persons (deliverable 3) and a list of conditions and main features of quality of life and/or services, as defined by older persons (deliverable 4). Following the presentation of the findings, some themes will be selected by the study partners to be further investigated in the following step, within the framework of a creativity workshop.

#### Step 2: Creativity workshop

This creativity workshop will consist in developing organisational or service adjustments, incremental and/or disruptive innovations in order to acknowledge and support what is important to older persons.

One hypothesis could be that housing pathways (anticipating and better planning the conditions for accommodation changes) could become the subject of a joint reflection between partners representing main healthcare and service providers for older persons in France (one of the main retirement funds in charge of autonomy loss prevention and home assistance for older persons, an insurance group in charge of providing complementary and innovating services to their clients, a hospital group with a nursing home network) in order to improve transitions, prevent older persons from giving up on activities and relationships that are important to them, and capitalize on opportunities associated with having a good life at home or in institutions.

We will limit ourselves to describing the workshop’s general and provisional framework as the workshop’s organisation and the co-creation methodology will depend on the themes chosen.

It will take place in 2 parts, in 2 complementary sessions:

- The first with the study partners, representatives of health and service providers.
- The second with health and service professionals, older persons met during Step 1 in nursing homes, independent-living facilities, at home and atypical accommodation.

The order of these sessions will be determined according to the co-creation strategy adopted, which will consist of either a direct exploration of the themes by the study partners whose insights and proposals will then be submitted to a panel of end-users (professionals and older persons) or, conversely, direct mobilisation of the end-users to initiate creativity work on the themes chosen by the study partners, who will then collectively further develop findings.

#### Step 3: Dissemination of findings in the regions under study and within the scientific community

Three panel discussions open to older persons, the general public and the various actors in the Silver Economy will be organised with the support of the study partners and their local correspondents in the main regions under study. Between 70 and 100 attendees are expected to attend each conference.

These panel discussions will have the specific aim of mobilising other public bodies (regions, departmental councils, cities and the CNSA (National Solidarity Fund for Autonomy) and raising their awareness about the value of this approach and the possibility of integrating it into their respective fields of intervention.

The findings of this project will also be promoted through conventional scientific channels (publications in Medline-indexed journals).

## Patient and public involvement

As outlined above, this is an action research in which the patients and public concerned are actors in the research, and shape the findings. In addition, in step 3 of the project, three panel discussions will be organized, which will be open to older persons, the general public and the various actors in the Silver Economy with the support of the study partners.

## Dissemination of results

The plan for dissemination of findings is consubstantial to the nature of this action research, which is based on a co-creation approach with older persons, health and service providers partners of this survey, and on sharing of results, both nationally and with other public actors in the regions under study (see structure of the project above, stages 1, 2, 3). As outlined in Step 3 of the project, panel discussions will be organized, which will be open to older adults, the general public and actors in the Silver Economy. These discussions will provide a public platform for presentation of the findings to interested parties, and will also be an opportunity to discuss the findings and how they can move practice forward. De-identified research data will be made publicly available when the study is completed and published.

## Discussion

This project will open a discussion addressing eudemonic well-being. Rather than aiming to contribute to existing general questions regarding perception of the meaning of life among older persons, this project will aim to offer points of reference for the content of the meaning of life through a typology of logics of action which structure, guide and motivate older persons in their daily life: being with others (being part of a group, being the centre of a group), keeping distance with others (being free), helping, or escaping. In this way, this project will not only contribute to our reflection on the various ways in which older persons give meaning to their daily life, but also on how these meaningful elements can be integrated into support services and policies for older persons.

This project will also revisit the principles underpinning existing quality of life questionnaires applied to older persons, particularly the WHOQOL OLD [35, 36], the CASP-19 [37] and the OPQOL [18]. A new look will be taken at the conditions of hedonic well-being for older persons, by targeting the essential conditions for quality of life, questioning the meaning of “being at home” and comparing the conditions of quality of life in a variety of accommodation types (at home, independent-living facilities, nursing homes and atypical housing). This comparison will also contribute to the reflection on housing pathway issues, and the optimal and diverse conditions for feeling “at home”.

### Study status

Step 1 of the study is under way. Interviews were performed with a panel of 20 nursing home residents in April and May 2021. A second panel of 20 autonomous older individuals in assisted living facilities are being interviewed between June and September 2021, and a third panel of 20 community-dwelling older individuals will be interviewed from September to November 2021. In parallel, a panel of 7 older individuals living in atypical housing types are being recruited and interviewed. Steps 2 and 3 will be launched once the data from Step 1 have been collected and analysed.
